# Liquid and vapour-phase antifungal activities of selected essential oils against *candida albicans*: microscopic observations and chemical characterization of *cymbopogon citratus*

**DOI:** 10.1186/1472-6882-10-65

**Published:** 2010-11-10

**Authors:** Amit K Tyagi, Anushree Malik

**Affiliations:** 1 Applied Microbiology Laboratory, Centre for Rural Development & Technology, Indian Institute of Technology Delhi, New Delhi- 110 016, India

## Abstract

**Background:**

Use of essential oils for controlling *Candida albicans *growth has gained significance due to the resistance acquired by pathogens towards a number of widely-used drugs. The aim of this study was to test the antifungal activity of selected essential oils against *Candida albicans *in liquid and vapour phase and to determine the chemical composition and mechanism of action of most potent essential oil.

**Methods:**

Minimum Inhibitory concentration (MIC) of different essential oils in liquid phase, assayed through agar plate dilution, broth dilution & 96-well micro plate dilution method and vapour phase activity evaluated through disc volatilization method. Reduction of *C. albicans *cells with vapour exposure was estimated by kill time assay. Morphological alteration in treated/untreated *C. albicans *cells was observed by the Scanning electron microscopy (SEM)/Atomic force microscopy (AFM) and chemical analysis of the strongest antifungal agent/essential oil has been done by GC, GC-MS.

**Results:**

Lemon grass (*Cymbopogon citratus*) essential oil exhibited the strongest antifungal effect followed by mentha (*Mentha piperita*) and eucalyptus (*Eucalyptus globulus*) essential oil. The MIC of lemon grass essential oil in liquid phase (288 mg/l) was significantly higher than that in the vapour phase (32.7 mg/l) and a 4 h exposure was sufficient to cause 100% loss in viability of *C. albicans *cells. SEM/AFM of *C. albicans *cells treated with lemon grass essential oil at MIC level in liquid and vapour phase showed prominent shrinkage and partial degradation, respectively, confirming higher efficacy of vapour phase. GC-MS analysis revealed that lemon grass essential oil was dominated by oxygenated monoterpenes (78.2%); α-citral or geranial (36.2%) and β-citral or neral (26.5%), monoterpene hydrocarbons (7.9%) and sesquiterpene hydrocarbons (3.8%).

**Conclusion:**

Lemon grass essential oil is highly effective in vapour phase against *C. albicans*, leading to deleterious morphological changes in cellular structures and cell surface alterations.

## Background

*Candida albicans *is the most common species associated with candidiasis and is the most frequently recovered species from hospitalized patients. Candidiasis encompasses infections that range from superficial, such as oral thrush [1] and vaginitis, to systemic and potentially life-threatening diseases. The increase of *C. albicans *infections parallels medical advancements such as invasive procedures, immunosuppressive treatments for organ transplants and widespread use of broad-spectrum antibiotics [2]. Excessive antibiotics use results in killing of the competing bacterial flora, leading to an over growth of yeasts [3,4].

The therapeutic approach to nosocomial infections is a great challenge due to the resistance developed by pathogens towards a number of widely-used drugs [5]. Therefore, the use of essential oils for the prevention and treatment of infection has been gaining popularity within the research field over the past decade [6,7]. Tea tree essential oil shows promise as a topical antifungal agent, with recent clinical data indicating efficacy in the treatment of dandruff [8] and oral candidiasis [9]. Data from an animal model also indicate that it may be effective in the treatment of vaginal candidiasis [10]. Recently, Karpanen *et al*. [11] demonstrated that chlorhexidine digluconate (CHG), eucalyptus essential oil, tea tree oil and thymol exhibit significant antimicrobial activity against *Staphylococcus epidermidis*. However, the concentration of essential oils required to achieve the same level of growth inhibition as CHG was several orders of magnitude higher (g/l for essential oils compared with mg/l for CHG). Nevertheless, essential oils at times may be more effective in controlling biofilm cultures due to their better diffusibility and mode of contact. For example, in the study by Al-Shuneigat *et al*. [12] staphylococci in a biofilm mode of growth demonstrated increased susceptibility to an essential oil-based formulation compared with planktonic cells. Karpanen *et al*. [11] also noticed that thymol showed increased activity against *S. epidermidis *growing in biofilm compared with planktonic cells. The authors suggested that being a phenolic compound, thymol has both hydrophilic and hydrophobic properties, which may enhance diffusion of this compound in a biofilm and allow its access to fungal cells where it alters the permeability of plasma membranes [13]. Hence, essential oils could be a better antimicrobial agent provided their efficacy is enhanced resulting in lower MICs.

Several approaches have been proposed to minimise essential oil concentrations. One of them is use of essential oils in vapour phase to reduce the required concentration. It is thought that the lipophilic molecules in the aqueous medium associate to form micelles and thus suppress the attachment of the essential oils to the organism, whereas the vapour state of the essential oils allows free attachment [14]. However, few studies are available on vapour phase antimicrobial activity of essential oils and these are concerned with cinnamon (*Cinnamon zeylanicum*), clove (*Syzygium aromaticum*), basil (*Ocimum basillicum*), rosemary (*Rosmarinus officinalis*), dill (*Anethum graveolens*), and ginger (*Zingiber officinalis*), essential oils [15,16]. Inouye *et al*. [17] investigated the antibacterial activity of 14 essential oils (including lemon grass, pippermint and eucalyptus essential oil) in gaseous phase against respiratory tract pathogens. However, to the best of the author's knowledge, no systematic studies comparing anticandidal activity (in liquid and vapour phase) of the lemon grass essential oil, mentha essential oil, or eucalyptus essential oil are available.

In the present work antimicrobial activity of lemon grass essential oil, mentha essential oils, and eucalyptus essential oil against the *C. albicans *has been observed in liquid as well as in vapour phase. To explain the antimicrobial efficacy of most potent lemon grass essential oil, chemical composition of this essential oil has been analysed by GC, GC-MS. Since one of the important factors underlying the virulence of fungi (and bacteria) is their ability to maintain the functional architecture of their envelopes, the aim of this study was also to investigate whether lemon grass essential oil acts by interfering with the envelope of *C. albicans*. Such studies have often been performed using optical and scanning electron microscopes, but in the present study along with SEM, atomic force microscopes (AFM) has been used. Recently, AFM has been employed for studying the effect of multimeric antimicrobial peptide SB006 on *Pseudomonas aeruginosa *[18] and that of thymol on *Candida albicans *[19]. To the best of author's knowledge, reports depicting application of AFM to investigate alterations of *C. albicans *cells by essential oil in liquid as well as vapour phase are highly scanty.

## Methods

### Chemicals and Strains

The essential oils were procured from Natural Aromatics Private Limited, New Delhi (India) and stored in air-tight sealed glass bottles at 4°C till further use. Growth media and Tween 80 were purchased from Himedia and Qualigens (India), respectively, while ethanol was purchased from Merck, India *Candida albicans *ATCC 10231 strain was collected from the central microbial culture facility, Department of Biotechnology & Biochemical Engineering, Indian Institute of Technology Delhi, New Delhi, India and used to evaluate the effect of essential oils.

### Inoculum preparation

The strain of *C. albicans *used in this study was grown in Potato Dextrose broth (PDB) medium at 30°C for 24 h in an orbital shaking incubator at 180 rpm. Cells were harvested by centrifugation, suspended in sterile distilled water and used immediately.

### Antimicrobial assays

#### Determination of MIC by agar dilution method

Minimum Inhibitory Concentration (MIC) of essential oils was determined by agar dilution assay. The agar plates were prepared using Yeast Potato Dextrose agar (PDA) (15 ml per petri dish) amended with various concentrations of plant essential oils (i.e. 270-18000 mg/l). For enhancing the essential oil solubility, Tween-80, 0.5% (v/v) was added. These plates were inoculated with one ml cell suspension (10^6 ^cfu/ml), of *C. albicans*. All the plates were incubated in triplicate for each concentration at 30°C for 48 h. Plates with Tween-80 but without any plant essential oil were used as control. Observation of the plates (fungal growth) was done at a time interval of 12 h up to 48 h of incubation. The MIC values were determined as the lowest concentration of essential oil preventing visible growth of *C. albicans *[20].

#### Determination of MIC and MFC using broth dilution method

Minimum fungicidal concentration (MFC) of essential oil was determined according to Broth Macro Dilution Assay [21]. A range of essential oil concentrations (270-18000 mg/L) was prepared in Yeast Potato Dextrose broth (PDB) medium. To enhance essential oil solubility, Tween-80 was included at a final concentration of 0.5% (v/v). Each flask was inoculated with 10^6 ^cfu/ml of the *Candida *strain. Flasks containing only Tween-80 (without plant essential oil) were used as control. The flasks were incubated at 30°C, in an orbital shaking incubator (180 rpm) for 48 h. One ml of culture was taken from each flask (where growth was not observed) for serial dilution to make the inoculum of 10^6 ^cfu/ml and inoculated on PDA plates and incubated at 30°C for 48 h. The plates were observed and MFCs were determined.

#### Colorimetric method for determination of inhibitory and fungicidal concentration of essential oils

The Essential oils which exhibited the antimicrobial activity were further tested to determine the concentrations at which they were fungistatic and fungicidal using a colorimetric broth micro-dilution technique [22]. In order to test concentrations from 144-18000 mg/l, sterile 96-well microplates with lid were set up as follows: in wells in row A were placed 200 μl portions of 18000 mg/l essential oil in sterile PDB; wells in rows B to H received 100 μl of sterile PDB. Serial two fold dilutions were carried out from row A to row H and excess broth (100 μl) was discarded from row H. To each well was added 100 μl of inoculum and Resazurin dye. The inoculum was prepared using a 20 h growing culture and further diluted with PDB to achieve approximately 10^6 ^cfu/ml. A positive control (containing inoculum but no essential oil) and negative control (containing essential oil but no inoculum) were also included. The contents of the wells were mixed and the microplates were incubated at 30°C for 24 h. A colour change from pink to purple was indicative of fungal growth.

Now an aliquot of 5 μl from the wells remaining pink were plated onto PDA and incubated for 24 h at 30°C. Two replicates of each microassay were carried out and the experiment was carried out twice. The fungistatic concentration was determined as the lowest concentration at which *C. albicans *at least three of the four replicates failed to grow in PDB but were cultured when plated onto PDA. The fungicidal concentration was the lowest concentration at which *C. albicans *in at least three of the four replicates failed to grow in PDB and were not cultured after plating onto PDA.

#### Disc volatilisation assay

Standard experimental set-up as described by Lopez et al. [15] was used. Briefly, a 100 μl portion of a *C. albicans *suspension containing approximately 10^6 ^cfu/ml was spread over the surface of a PDA plate and allowed to dry. A paper disc (diameter 6 mm, Sigma Aldrich) was laid on the inside surface of the upper lid and 10 μl essential oil was placed on each disc. The plate inoculated with *C. albicans *was immediately inverted on top of the lid and sealed with parafilm to prevent leakage of the vapour. Plates were incubated at 30°C for 24 h and the diameter of the resulting inhibition zone in the fungal lawn was measured. Volume of essential oils tested was varied (20, 40 or 60 μl) by using appropriate number of sterile discs.

### Determination of the kill time

These experiments were conducted for selected efficient essential oil vapours in a compact chamber made up of acrylic material (size 50 cm × 50 cm; W× L). The height of the chamber was 50 cm on the back side and 25 cm at the front side. The total volume of the chamber was 0.09375 m^3 ^(93.75 l). The front side of the chamber had gloves through which the things inside the chamber could be handled without opening the chamber. Prior to exposure the chamber was cleaned with ethanol and UV sterilized. Two essential oil evaporating machine (Khera instruments Pvt. Ltd, New Delhi, India; evaporation rate = 0.50 ml/h) were fixed in this chamber as described earlier [23]. Appropriate serial dilution of the culture (to obtain 100-300 cfu) was plated on PDA plates. After a particular time period (0.5, 1, 2, 4, and 8 h) the plates were detached, closed and incubated at 30°C for 18 - 20 h. All the plates were used in triplet.

### Preparation of C. albicans samples for morphological study

The *C. albicans *cells were incubated for 14 h in PDB at 30°C and 180 rpm. The suspension was divided into two portions. In one portion, suitable concentration of the essential oil was added and another portion was left untreated as a control. The resuspension was incubated at 30°C for 4 h, and then the cells from both tubes were harvested by centrifugation and were prefixed with a 2.5% glutaraldehyde solution overnight at 4°C. After this, the cells were again harvested by centrifugation and washed three times with 0.1 M sodium phosphate buffer solution (pH 7.2). Now each resuspension were serially dehydrated with 25, 50, 75, 90, and 100% ethanol, respectively. Then, cells were dried at "critical point" [24].

For SEM, a thin film of cells was smeared on a silver stub. The samples were gold-covered by cathodic spraying (Polaron gold). Finally, morphology of the *C. albicans *cells was observed on a scanning electronic microscope (ZEISS EVO 50). The SEM observation was done under the following analytical condition: EHT = 20.00 kv, WD = 9.5 mm, Signal A = SE_1_.

The AFM images were taken employing the Veeco Metrology Group of nanoscope IIIa operating in contact mode. In this mode of operation, a silicon nitrite tip with a force constant of 0.58 N/m was used. For AFM mounting of *Candida *cells, glass substrates were employed. Ten micro litres of each lemon grass essential oil treated, lemon grass essential oil vapour treated and untreated *Candida *cells suspension was mounted on a glass substrate. After air-drying the cells were imaged in air with AFM in tapping mode.

### Gas chromatographic (GC) and Gas chromatographic mass spectrometry (GC-MS) analysis

The percentage composition of essential oil was determined by GC-FID and the compounds were identified by GC-MS. GC analysis was carried out on a Shimadzu 2010 Gas Chromatograph equipped with an FID and 25 m × 0.25 mm × 0.25 μm WCOT column coated with diethylene glycol (AB-Innowax, 7031428, Japan). Both injector and detector (FID) temperatures were maintained at 260°C. Helium was used as carrier gas at a flow rate of 3.0 ml/min at a column pressure of 152 kPa. Samples (0.2 μl) were injected into the column with a split ratio of 100:1. Component separation was achieved following a linear temperature program of 60 - 260°C at 3°C/min and then held at 260°C for 10 min, with a total run time of 76 min. The percentage composition was calculated using peak normalization method assuming equal detector response. The samples were then analysed on same Shimadzu instrument fitted with the same column and following the same temperature program as above. MS parameters used were; Ionisation Voltage (EI) 70 eV, peak width 2 s, mass range 40-600 amu and detector voltage 1.5 Volts. Results are based on GC-FID; MS acquisition started after 4 min. Peak identification was carried out by comparison of the mass spectra with mass spectra available on database of NIST05 and WILEY8 libraries and co-injection of available pure standards. The compound identification was finally confirmed by comparison of their relative retention indices with literature values [25].

### Statistical analyses

All experiments were repeated at least twice. Data were analysed by Using SPSS (version 10) statistical software. Effect of treatments on *C. albicans *were analysed using one way ANOVA. Duncan multiple range test was used to compare the significance of differences among treatments at *P *values of < 0.05.

## Results

### Determination of MIC and MFC of essential oils against C. albicans

#### Agar plate dilution and broth dilution Method

MICs of the three essential oils were determined against *C. albicans*. The essential oils exhibited concentration-dependent inhibition of growth. A 288 mg/l concentration of lemon grass essential oil was enough for complete growth inhibition of *C. albicans *while mentha and eucalyptus essential oil required 1125 mg/l and 1750 mg/l essential oil concentration, respectively.

Minimum fungicidal concentration (MFC) is defined as the lowest concentration of essential oil resulting in the death of 99.9% of the inoculum [20]. All the essential oils inhibiting growth showed fungicidal activity. In general, it has been observed that the MFC was higher than MIC (Table 1).

**Table 1:** MICs and MFCs of mentha essential oil, eucalyptus essential oil and lemon grass essential oil obtained by different methods

Essential oils	Agar plate dilution Method		Broth dilution method		96-well microplate method	
	
	MIC (mg/ml)	MFC (mg/ml)	MIC (mg/ml)	MFC (mg/ml)	MIC (mg/ml)	MFC (mg/ml)
**Mentha oil**	1.125	2.25	1.125	2.25	1.125	2.25

**Eucalyptus oil**	2.25	4.5	2.25	4.5	2.25	4.5

**Lemon grass oil**	0.288	0.567	0.567	1.125	0.288	0.567

#### Determination of MIC/MFC of essential oils with 96-micro well plate method

The fungistatic and fungicidal concentrations of essential oils obtained by colorimetric assay followed by plating out on PDA are presented in Table 1. As observed in the previous assays, lemon grass essential oil exhibited the strongest antifungal effect followed by mentha essential oil and eucalyptus essential oil.

#### Zone of inhibition due to the essential oil vapours

**Figure 1:**
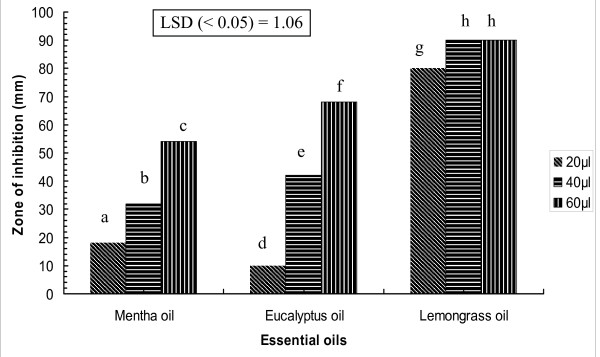
**Zone of inhibition due to essential oil vapours of mentha essential oil, eucalyptus essential oil and lemon grass essential oil at different concentrations.** The bar of treatment followed by same letter did not differ significantly by Duncan multiple range test (DMRT; P = 0.05); LSD, least significant difference by ANOVA.

The zone of inhibition resulting from the exposure to different essential oil vapours varied due to the presence of different volatile chemical components. It also increased with increasing concentration of the essential oil (Figure 1). Zone of inhibition due to vapours generated by 20 μl lemon grass essential oil was higher (i.e. 80 mm) than eucalyptus (10 mm) and mentha essential oil (18 mm). Vapours from 40 μl of lemon grass essential oil were enough to completely inhibit the growth of *C. albicans *while any other essential oil vapour was not able to achieve the same upto 60 μl. Since lemon grass essential oil showed the highest MIC in liquid phase and greater zone of inhibition in vapour phase, this was selected for further study.

### Kill time assay

**Figure 2:**
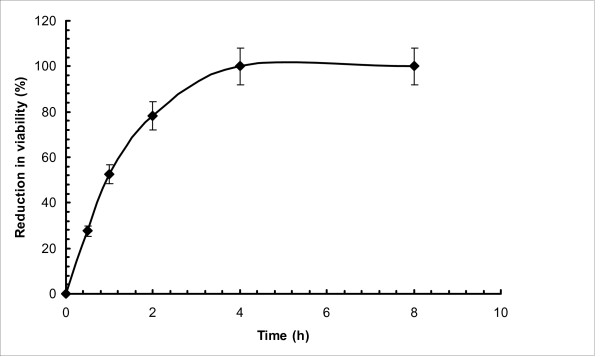
**Kill Time Assay; percentage reduction in viability of *C. albicans *due to pre-incubation exposure to lemon grass essential oil vapour for different time durations**.

Further experiments were conducted to validate the efficacy of lemon grass essential oil vapour in terms of kill time of *C. albicans *by exposing the inoculated plates to lemon grass essential oil vapour in the closed airtight chamber for 8 h. Result of this study are shown in Figure 2. During the initial period, significant reduction in viability (i.e. 52.48% in one hour) of *C. albicans *was observed. Within 4 h duration, 100% reduction in viability had been observed.

### Morphological alteration of C. albicans

#### Scanning electron microscope (SEM) observation

**Figure 3:**
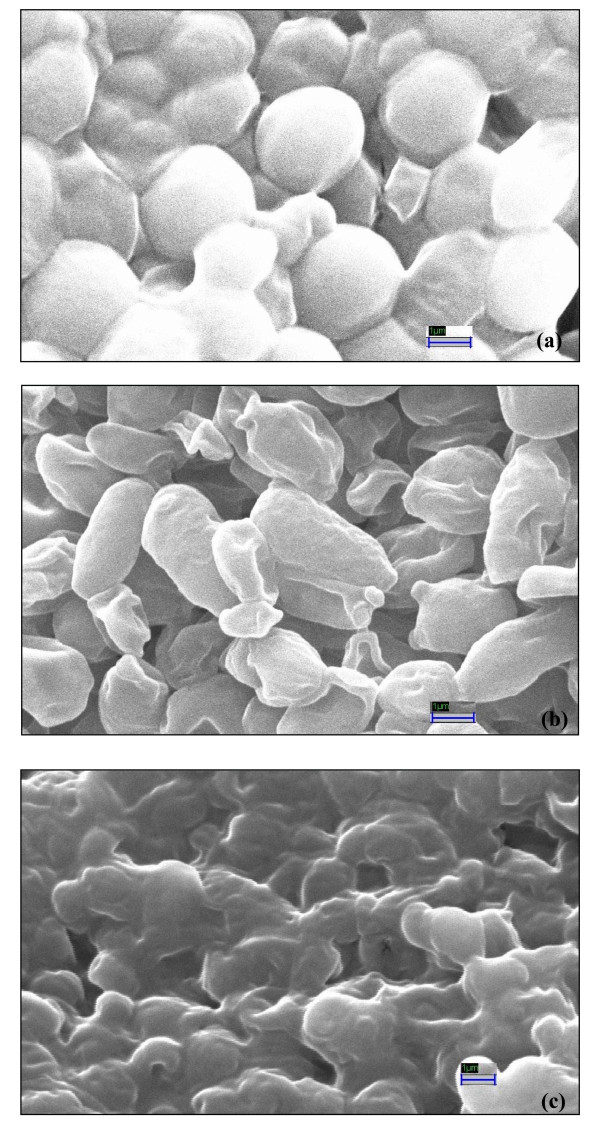
**Scanning electron micrographs of untreated and treated (24 h) *C. albicans *cells: (a) Untreated cells with normal smooth surfaces (× 25.00 K), (b) Shrinked and deshaped lemon grass essential oil treated cells (× 25.00 K), (c) Non-uniform/deformed and ruptured lemon grass essential oil vapour treated cells (× 25.00 K)**.

Figure 3 shows the untreated and lemon grass essential oil (liquid and vapour phase) treated *C. albicans *cells. Cells treated with lemon grass essential oil at MIC level underwent considerable morphological alterations in comparison to the control when observed by a Scanning Electron Microscope (Figure 3). Control cells appeared turgid and whole (Figure 3a) while the lemon grass essential oil (288 mg/l) treated cells appeared to be empty of contents and shrunken (Figure 3b). The cells were completely destroyed when exposed to 32.7 mg/l of lemon grass essential oil vapour (Figure 3c). Hence change in morphology and destruction of the *C. albicans *cells appeared more in vapour phase exposure than the broth phase treatment.

#### Atomic force microscope (AFM) observation

**Figure 4:**
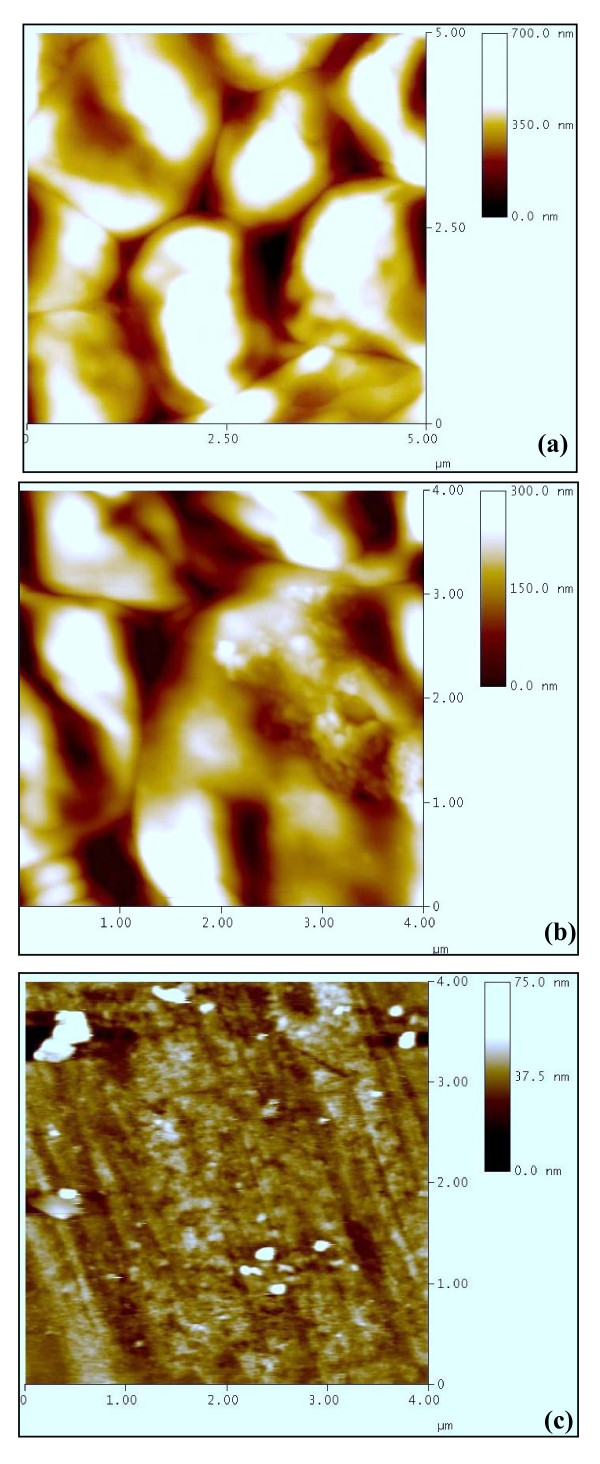
**Atomic force micrographs showing variation in the height of untreated and treated (24 h) *C. albicans *cells from glass surface: (a) Untreated (h 350 nm), (b) Lemon grass essential oil treated (h 150 nm), (c) Lemon grass essential oil vapour treated (h 37.5 nm)**.

The AFM picture of untreated cells (Figure 4a) show cluster of *C. albicans *cells. The clustering is similar to what has been observed with SEM. Untreated sample clearly shows the intact shape of the *C. albicans *cells. In the lemon grass essential oil treated cells (Figure 4b), cells loose their original shapes, appear shrunken and partially deformed. Although length and width seems to be less affected, cells appear relatively flattened. The lemon grass essential oil vapour treated *C. albicans *cells become strongly fragmented and completely lose their solidarity (Figure 4c). An outstanding aspect of AFM is the fact that it is possible to obtain a cross-section of the image and measure the height and size of the observed features precisely. Significant variations in the height of cells have been found in this study. The height of untreated, lemon grass essential oil treated and lemon grass essential oil vapour treated cells was found to be 350 nm (Figure 4a), 150 nm (Figure 4b) and 37.5 nm (Figure 4c), respectively.

**Figure 5:**
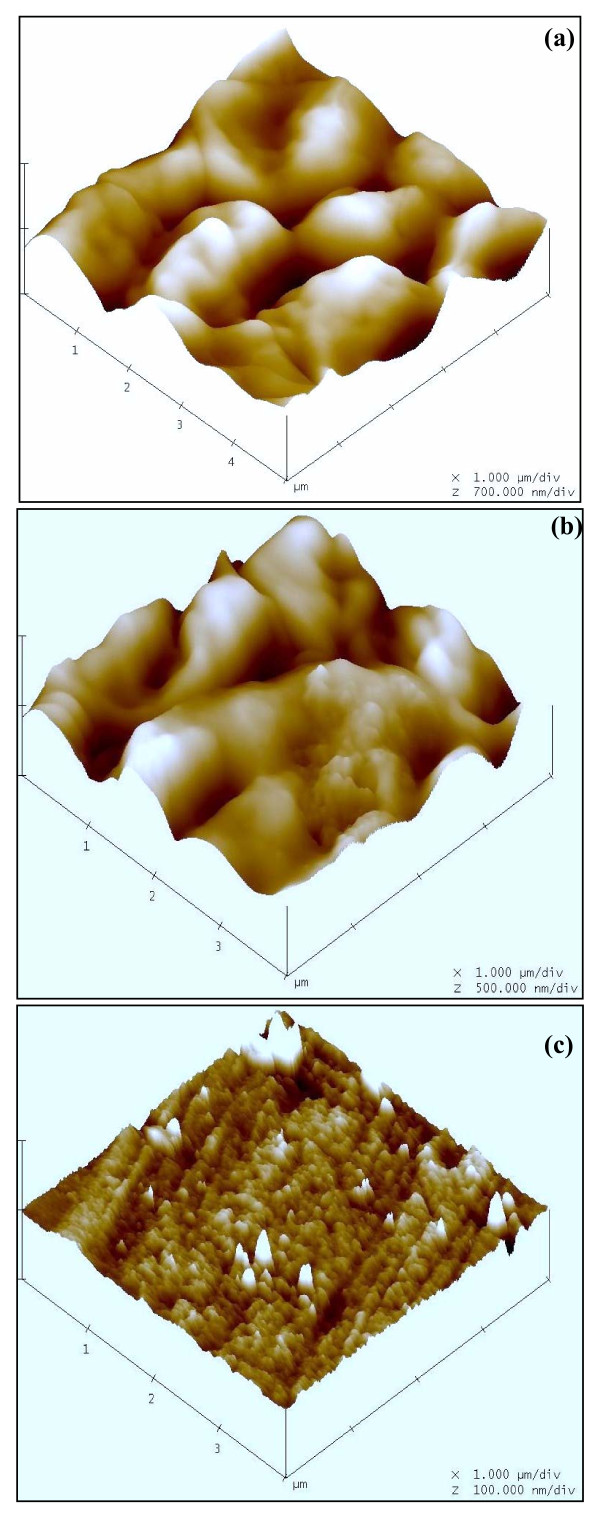
**Atomic force micrographs showing three dimensional view of *C. albicans *cells: (a) Untreated (z 700 nm/div), (b) Lemon grass essential oil treated (z 500 nm/div), (c) Lemon grass essential oil vapour treated (z 100 nm/div)**.

Atomic force microscopes simultaneously measure surfaces in the *x, y*, and *z *dimensions and provide a true three-dimensional map of the surface of cell samples on a submicron scale. The three dimensional structure of the *C. albicans *cells also shows significant differences in the Z axis value which was 700 nm/div (Figure 5a), 500 nm/div (Figure 5b) and 100 nm/div (Figure 5c) in untreated, lemon grass essential oil treated and lemon grass essential oil vapour treated samples, respectively. Highly irregular and rough surface structure observed in case of vapour treated cells represents complete rupture and loss of structure in vapour treated cells. It is clear from these results that the vapour treatment not only perceptibly alters the cell dimensions and the overall morphology, but a great impact on the cell surface properties is also noticed.

### Chemical characterisation of the essential oil constitutes

Qualitative and quantitative analysis of the lemon grass essential oil is listed in Table 2. In lemon grass essential oil, 37 components were identified, which represented about 94.5% of the total detected constituents. The essential oil contains a complex mixture consisting mainly monoterpene hydrocarbons (7.9%), oxygenated monoterpenes (78.2%), sesquiterpene hydrocarbons (3.8%) and oxygenated sesquiterpenes (1.6%). A portion (5.5%) of total composition was not identified. The major constituents of the essential oil were α-citral or geranial (36.2%), β-citral or neral (26.5%), Nerol (5.1%), limonene (4.19%), Neryl acetate (4%) and 5-hepten-2-one (2.9%). Other components were present in amounts less than 2%.

**Table 2:** Chemical composition of lemon grass essential oil:

RT (min)	Compound	Percentage	RI
6.2	Tricyclene	0.2	1009

6.4	Pinene	0.4	1036

7.1	Camphene	1.5	1066

8.6	3-carene	0.1	1141

9.0	β-myrcene	0.8	1156

9.9	Limonene	4.2	1206

11.2	β-ocimene	0.3	1228

11.4	Cineole	0.2	1233

11.7	β-ocimene	0.4	1250

12.3	n-octanal	0.1	-

13.7	6-methyl-hepten-2-one	2.9	-

16.3	Myrtanal	0.2	-

18.0	β-Citronellal	0.7	1465

19.8	Linalool	1.8	1506

21.7	β-Caryophyllene	1.9	1533

24.1	β-Citral	26.5	1680

24.2	Sabinol	0.5	1683

24.4	α-cyclocitral	0.5	-

24.5	Borneol	0.2	1698

24.6	Neryl acetate	4.0	1699

24.9	Germacrene-D	0.5	1712

25.2	β-citronellol	0.9	1722

25.5	Zingiberene	0.1	1728

25.6	α-Citral	36.2	1730

25.7	Verbenone	0.2	1733

26.1	Nerol	5.1	1757

26.3	γ-cadinene	1.3	1766

28.2	Z-carveol	0.2	1820

29.7	Geranial butyrate	0.2	1872

32.5	Caryophyllene oxide	0.8	1966

34.0	Epi-Cubenol	0.3	2037

45.2	Isoeugenol	0.5	-

46.3	Nerolic acid	0.8	-

	Monoterpene hydrocarbons	7.9	

	Oxygenated monoterpenes	78.2	

	Sesquiterpene hydrocarbons	3.8	

	Oxygenated sesquiterpenes	1.6	

	Total of identified compound	94.5	

## Discussion

The investigations on antimicrobial activity of three essential oils against *C. albicans *in liquid phase showed that lemon grass essential oil had higher activity as compared to mentha and eucalyptus essential oil. The MIC of lemon grass essential oil was 288 mg/l while that for mentha and eucalyptus essential oil was 1125 mg/l and 2250 mg/l, respectively. Interestingly, the MIC and MFC values obtained by all the antimicrobial assays (Agar plate dilution method, 96- well microplate method and broth dilution method) were similar except that the broth dilution method gave higher MIC for lemon grass essential oil. The MFC of the essential oils followed the same trend i.e. lemon grass essential oil (567 mg/l) < mentha essential oil (2250 mg/l) < eucalyptus essential oil (4500 mg/l).

The antimicrobial activity of essential oils, plant extracts and pure components against *C. albicans *has been reported earlier also. The MIC values obtained in the previous studies are shown in Table 3. The MIC for lemon grass essential oil obtained in the present study (288 mg/l) is substantially lower than that reported earlier for mentha essential oils, *Pinus desiflora, Cassia spectabilis*, Tea tree essential oil or even the pure active compounds like 1-8-cineole, p-cymene etc. It is also better than the earlier reports on MIC of lemon grass essential oil for *C. albicans*, where the MICs ranged from 0.06-0.12% (Table 3) [19,26-36].

**Table 3:** MIC of different essential oil/chemical component for *C. albicans*

**S.N**.	Name of essential oil/chemical component	MIC value for *C. albicans*	Reference
**1**.	*Coriandrum sativum*	163 mg/l	[26]

**2**.	*Satureja biflora*	950 mg/l	[27]

**3**.	*Satureja masukensis*	1190 mg/l	[27]

**4**.	*Chaerophyllum libanoticum*	250 - 500 mg/l	[28]

**5**.	*Cympobogon winterianus*	600 mg/l	[29]

**6**.	*Mentha piperita*	600 mg/l	[29]
**7**.	*Mentha pulegium*	7400 mg/l	[29]

**8**.	*Mentha arvensis*	7400 mg/l	[29]

**9**.	*Lippia sidoides*	620-2500 mg/l	[30]

**10**.	*Pinus desiflora*	2180 mg/l	[31]

**11**.	*Cassia spectabilis*	6250 mg/l	[32]

**12**.	*Tanacetum argenteum*	125 mg/l	[33]

**13**.	*Ferula glauca*	1250 mg/l	[34]

**14**.	*Coriandrum sativum*	163000 mg/l	[35]

**15**.	*Tarchonanthus camphoratus*	113000 mg/l	[35]

**16**.	Thymol	125 mg/l	[19]

**17**.	Pinene	310 mg/l	[34]

**18**.	(E)-caryophyllene	155 mg/l	[34]

**19**.	caryophyllene oxide	78 mg/l	[34]

**20**.	Eugenol	500 mg/l	[19]

**21**.	Camphor	4850 mg/l	[27]

**22**.	α-Pinene	4000 mg/l	[27]

**23**.	Linalool	125 mg/l	[36]

**24**.	α-Terpineol	500 mg/l	[36]

The relative antimicrobial efficacy of essential oil vapours (measured as the inhibition zone) also showed the same trend as in the liquid phase assay (i.e. lemon grass essential oil < mentha essential oil < eucalyptus essential oil) at lower concentration tested (20 μl essential oil or 16.6 mg/l of air). However at higher concentration (40 and 60 μl corresponding to 32.7 and 65.4 mg/l of air), eucalyptus essential oil vapour caused higher zone of inhibition as compared to mentha vapours. Lemon grass essential oil completely inhibited the growth as evident by the 90 mm dia of the inhibition zone, although other essential oils could not evoke such complete inhibition. Hence, based on the disc volatilisation assay, the MIC of lemon grass essential oil in vapour phase (32.7 mg/l) was significantly lower than that in the liquid phase (288 mg/l). In the direct contact assays for liquid phase, the activity depends upon the diffusability and solubility of the essential oil compounds into the agar while the antimicrobial activity of the vapour assay depends upon the volatility of each compound [16]. Since active compound of essential oils are highly volatile, therefore, essential oils possess high antimicrobial activity in vapour phase. Inouye *et al*. [17] tested the activity of 14 essential oil in gaseous phase against respiratory tract pathogens. They also observed that minimum inhibitory dose of lemon grass essential oil was significantly lower than peppermint essential oil and eucalyptus essential oil in gaseous phase.

The results of the present study can be explained on the basis of chemical composition of essential oils. The major active component in *C. citratus, M. piperita *and eucalyptus essential oils used in the present study is citral, menthol and 1-8,cineole, respectively (Unpublished data). Earlier, Moleyar and Narasimhan [37] evaluated the antifungal activity of pure essential oil components in liquid culture. They observed that unsaturated aldehyde like citral and cintronellal were most active and the MIC followed the following trend: citral (100 mg/l) < menthol (200 mg/l) < eucalyptol (500 mg/l). Similarly, the growth inhibition produced by citral vapours was significantly higher than that produced by menthol vapours [37]. Inouye *et al*. [17] also confirmed that vapours of aldehyde constituents like cinnamaldehyde and citral were most potent while terpene alcohols (menthol) showed moderate activity and the terpene ketones and terpene ethers (1,8-cineole) were very weak antimicrobial agents. In the present study, lemon grass essential oil showed best performance both in liquid as well as vapour phase. Hence detailed chemical characterization of this essential oil was done by GC-MS. The results confirmed that the highest antifungal activity of lemon grass essential oil recorded here could be correlated to the presence of high level of oxygenated monoterpenes (78.2%) constituted by geranial (α-citral) and neral (β-citral) as its major components. Citral is a mixture of two isomers, geranial and neral, which are acyclic α, β-unsaturated monoterpene aldehydes [38,39] and as discussed earlier possess significant antimicrobial activity. Apart from this, lemon grass essential oil contained monoterpene hydrocarbon (7.9%) such as camphene (1.5%) and limonene (4.2%). The antimicrobial action of monoterpenes suggests that they diffuse into and damage cell membrane structures [40]. It is known that the antimicrobial action of such molecules depends on their presence in gaseous form facilitating their solubilization in cell membranes [36]. Therefore, higher cell damage is expected to occur in lemon grass essential oil vapour treated cells. To confirm this hypothesis and to clarify the mechanism of action of lemon grass essential oil in liquid and vapour phase, SEM and AFM was employed.

Application of SEM and AFM for high spatial resolution surface imaging and morphology analysis of fungal cells has been recommended as a useful tool [41]. AFM is theoretically capable of higher resolution imaging than SEM and delivering the quantitative results. It also avoids the need for vacuum conditions or the coating of surfaces with layers of metal that may interact with samples and cover their fine natural structure. Therefore, AFM reveals the real roughness of the surface of the cell envelope, which other types of microscopy frequently show as being relatively smooth. In the present study, majority of the *C. albicans *cells treated with lemon grass essential oil at MIC level in broth show prominent shrinkage in the SEM. A more extensive damage is observed in *C. albicans *cells treated with lemon grass essential oil vapour which appear ruptured and partially degraded. These finding have been justified by AFM studies. Additionally, the information obtained on the height of the cells also confirmed the rupturing and flattening of cells upon exposure to lemon grass essential oil vapours. The three dimensional AFM images demonstrated extensive damage and considerable increase in cell surface roughness in lemon grass essential oil vapour treatment. This dramatic change in morphology differs from the alteration of surface roughness conserving the original bacterial shape observed by Da Silva *et al*. [42] and Meinken *et al*. [40] whereas it resembles the extensive cell damage observed by Braga and Ricci [43] and Li *et al*. [44]. Our AFM observations show that major surface alterations and deformities accompany the membrane damage induced by lemon grass essential oil vapours. These results are supported by the observation that terpenes alter cell permeability causing changes in membrane properties and functions by increasing membrane fluidity and altering membrane permeability [19].

Abe *et al*. [45] reported that the lemon grass essential oil (100 mg/l) and citral (25-200 mg/l) significantly inhibited the mycelial growth of *C. albicans*, suggesting the potential value of lemon grass essential oil for the treatment of cutaneous candidiasis. Fontenelle *et al*. [30] suggested that *Lippia sidoides *essential oil with MICs ranging from 620 - 2500 mg/l against different strains of *Candida *spp. could be a promising source of antifungal drug. Nevertheless, these results as well as several other reported MICs of herbal constituents for *Candida *(Table 3) indicate that for posing stringent competition to existing drugs, the MICs of herbal products must be lowered. In this regard, the MIC of lemon grass essential oil vapour (32.7 mg/l) obtained in the present study indicates that lemon grass essential oil in vapour phase could be a very effective antimicrobial agent for prevention the *C. albicans *growth. Nevertheless, in order to develop the application further, it is necessary to work out the appropriate exposure time for achieving complete disinfection or killing. Therefore, kill time assay has been conducted upto 8 h in the closed chamber. Reduction in viability of the *C. albicans *cells increased with the time upto 4 h. At 4 h exposure, 100% loss in viability of cells was observed. The calculated lemon grass essential oil vapour concentration at 4 hours was 38 mg/l of air. Hence, these results are also in agreement with those obtained via the zone of inhibition assay.

## Conclusion

It can be concluded that lemon grass essential oil vapour is more potent inhibitor of *C. albicans *growth, leading to deleterious morphological changes in cellular structures and cell surface alterations as compared to lemon grass essential oil. All of these phenomena leading to major surface alterations and deformities also reduce the ability of the fungi to adhere and consequently reduce their virulence and infectiousness. The use in vapour phase could have additional advantages such as efficacy without requiring direct contact resulting in ease of application. Further evaluation of the growth inhibition of *C. albicans *by lemon grass essential oil vapour *in vivo *is warranted.

## Competing interests

The authors declare that they have no competing interests.

## Authors' contributions

AKT carried out the study design, experimental part such as essential oil collection, inoculum preparation, antimicrobial evaluation, microscopic observation and chemical characterisation of lemon grass essential oil. AM supervised the work, evaluated the results, corrected the manuscript for publication and revised it critically. Both authors read and approved the final manuscript.

## Pre-publication history

The pre-publication history for this paper can be accessed here:

http://www.biomedcentral.com/1472-6882/10/65/prepub
